# Underexplored Molecular Mechanisms of Toxicity

**DOI:** 10.3390/jox14030052

**Published:** 2024-07-18

**Authors:** Olatunbosun Arowolo, Alexander Suvorov

**Affiliations:** Department of Environmental Health Sciences, School of Public Health and Health Sciences, University of Massachusetts, 686 North Pleasant Street, Amherst, MA 01003, USA; oarowolo@umass.edu

**Keywords:** publication bias, underexplored pathways, toxicity mechanisms

## Abstract

Social biases may concentrate the attention of researchers on a small number of well-known molecules/mechanisms leaving others underexplored. In accordance with this view, central to mechanistic toxicology is a narrow range of molecular pathways that are assumed to be involved in a significant part of the responses to toxicity. It is unclear, however, if there are other molecular mechanisms which play an important role in toxicity events but are overlooked by toxicology. To identify overlooked genes sensitive to chemical exposures, we used publicly available databases. First, we used data on the published chemical–gene interactions for 17,338 genes to estimate their sensitivity to chemical exposures. Next, we extracted data on publication numbers per gene for 19,243 human genes from the Find My Understudied Genes database. Thresholds were applied to both datasets using our algorithm to identify chemically sensitive and chemically insensitive genes and well-studied and underexplored genes. A total of 1110 underexplored genes highly sensitive to chemical exposures were used in GSEA and Shiny GO analyses to identify enriched biological categories. The metabolism of fatty acids, amino acids, and glucose were identified as underexplored molecular mechanisms sensitive to chemical exposures. These findings suggest that future effort is needed to uncover the role of xenobiotics in the current epidemics of metabolic diseases.

## 1. Introduction

The existing research demonstrates that the distribution of research efforts focusing on specific genes, proteins, and molecular mechanisms is biased by social factors that are not always related to the importance of corresponding molecules for living organisms. For example, the analysis of the temporal patterns of the publications on specific genes suggests that some genes received exceptional attention due to societal needs (cure for an important disease) [1]. Only a fraction of protein kinases, ion channels, G protein-coupled receptors, and nuclear receptors received significant research attention, not explained by their more important roles in cells [2,3,4].

Surprisingly, the list of proteins [3] and genes [5] which attracted most of the research attention did not change over the period of 20 years. A high level of research attention to a selected list of molecules may be partly explained by trends in the scientific community resulting from the dynamics of social networks [1]. These trends are successfully modeled based on vast publication data demonstrating that researchers predominantly publish on genes that are already popular in the research literature [6,7]. At least partly, these trends may originate from the social phenomena known as “famous for being famous” in popular culture. Additional social bias results from a catch-22 situation when research hypotheses are being developed using molecular pathways and functional biological categories based on well-annotated molecules. The resulting hypotheses focus further research efforts on the same set of molecules and pathways [8]. A recent analysis of gene features in association with the number of published studies showed that features that hinder our ability to study specific genes using traditional methodologies are associated with a smaller number of papers [5].

Taken together, these studies suggest that medico-biological research is prone to bias due to a broad range of factors, which all together concentrate the attention of researchers on a small number of well-known molecules/mechanisms leaving others underexplored. In accordance with this view, central to mechanistic toxicology is a narrow range of molecular pathways that are assumed to be involved in a significant part of toxicities. It is unclear, however, if there are other molecular mechanisms overlooked by previous research which also play a significant role in toxicity events. A recent attempt to identify in an unbiased manner molecular mechanisms most sensitive to a broad range of chemical exposures demonstrated that indeed a range of molecular mechanisms poorly covered by toxicological research may be as sensitive to toxins as those that received significant attention [9,10].

Toxicology started to use molecular biology tools in the late 1970s [11]; however, the rapid transformation into a molecular discipline was triggered by the publication of Toxicity Testing in the 21st Century: a Vision and a Strategy in 2007 by the US National Research Council [12]. One important component of this transformation consists of the understanding of molecular mechanisms that causally link exposures with adverse outcome–toxicity pathways or adverse outcome pathways (AOP) [12,13,14,15,16]. The characterization of AOP is critically important for the transition to pathway-based toxicity testing [17]. Thus, a systematic effort is needed to identify and characterize AOP and ensure that no important mechanisms linking exposures and toxicities have been overlooked.

In this report, we attempt to use minimally biased approaches to identify underexplored genes and molecular mechanisms sensitive to chemical exposures to inform the toxicological community on the important directions of future research.

## 2. Materials and Methods

### 2.1. Sensitivity of Genes to Chemical Exposures

Previous research developed an approach to identify genes sensitive to chemical exposures in an unbiased way [9,18]. In short, transcriptomic data were extracted from toxicological experiments in which gene expression changes in responses to chemical exposures were analyzed using high-throughput methods. Transcriptomic information from 2169 individual in vivo and in vitro studies using human, rat, or mouse cells or tissue covering experiments with 1239 chemical compounds was extracted from the Comparative Toxicogenomics Database (CTD) on 24 August 2018 (https://ctdbase.org) [19]. Genes that are not present in the genomes of all three species were excluded from further analysis. The number of published chemical–gene interactions (CGIs) was calculated for 17,338 genes to represent their sensitivity to chemical exposures. It is important to note that the ranked sensitivities of genes to chemical exposures do not depend on the composition of chemicals used for the identification of CGI numbers [9]. The full list of genes with their corresponding CGI numbers is available through Mendeley Data [20]. The threshold between the genes highly sensitive to chemical exposures and genes with low sensitivity was determined using a method for the identification of cutoff points in descriptive high-throughput omics studies [21]. This approach identifies an inflection point in a ranked distribution of variables if this distribution follows a biphasic pattern. In the current study, the method identified a cutoff between the big number of genes with low CGI numbers (<73) and the smaller group of genes with high CGI numbers (≥73). The identification of CGI was restricted to humans, mice, and rats as it required the consolidation of a big number of published datasets using the same gene IDs. Additionally, the inclusion of taxonomically distant organisms may generate subsets of genes (e.g., chemically sensitive genes) comprising genes that do not have homology in all the studied organisms. The identification of enriched molecular pathways for such subsets may be challenging.

### 2.2. Number of Publications Per Gene

The level of research attention for every human gene was evaluated by the number of PubMed publications that mentioned the gene in the title and/or abstract. This analysis was conducted by T. Stoeger’s group using PubTator [22]. As a result of this research, the authors created a database and a tool Find My Understudied Genes (FMUG, https://fmug.amaral.northwestern.edu/, accessed on 2 July 2024) which contains the number of publications per every human gene. This information for 19,243 genes was downloaded for our analysis. We used the same method for the cutoff point identification as described in the previous paragraph [21] to determine the threshold between the underexplored (<200 publications/gene) and well-explored (≥200 publications/gene) genes. The 200 publications per gene cutoff corresponds to the maximum inflection point in the biphasic distribution of publication numbers per gene for a ranked list of genes and delineates a big group of genes with small publication numbers and a smaller group of genes with big publication numbers.

### 2.3. Underexplored Pathways Sensitive to Chemical Exposures

To analyze biological categories enriched by underexplored genes sensitive and non-sensitive to chemical exposures, the list of all the genes with CGI number ≥ 73 or CGI < 73, respectively, were used with their respective publication numbers for gene set enrichment analysis (GSEA 4.3.3) [23,24]. GSEA was developed to characterize the cumulative shift of genes in a particular pathway towards an increase or decrease in expression. As such, it was designed for an input in which the values of gene expression changes have positive and negative values. To prepare datasets suitable for GSEA, in accordance with our threshold of 200 publications/gene, we subtracted 200 from the values of publications/gene to achieve negative publication values for underexplored genes and positive for well-explored genes. The resulting gene lists with publication values were uploaded to GSEA and analyzed against three independent databases: Reactome [25,26], KEGG [27], and Gene Ontology [28,29]. Additionally, the shortlist of top genes with the highest number of CGIs (CGI number ≥ 76) and the 10th percentile lowest number of publications (≤20 publications/gene) was uploaded to ShinyGO 8.0 [30] for the analysis of enriched biological categories, to DAVID 2021 [31,32] for the analysis of enriched clusters of functional annotations, and to Metascape 3.5 [33] for the analysis of DisGeNET [34] disease categories.

## 3. Results

**Figure 1:**
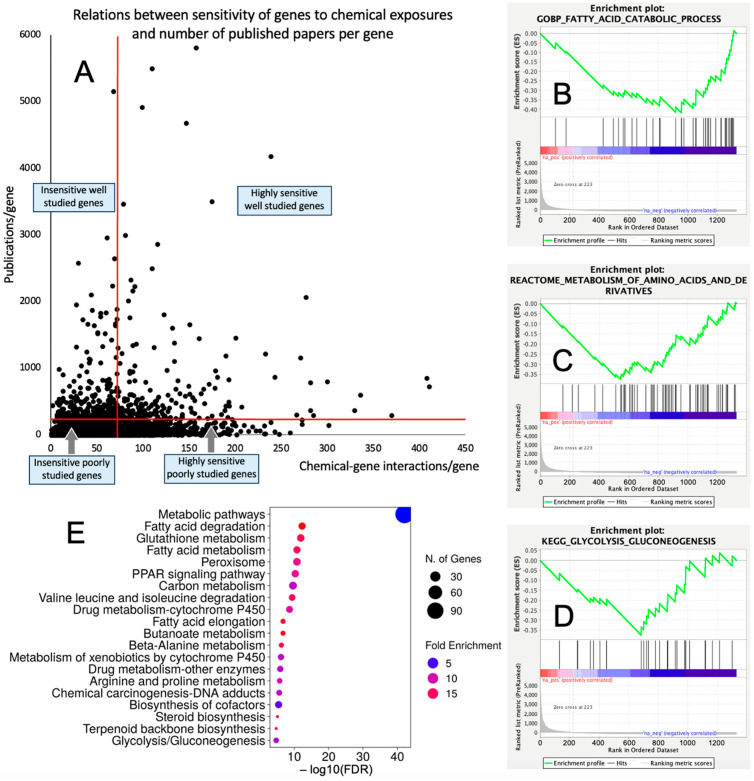
Underexplored molecular mechanisms of toxicity. (**A**) Distribution of 16,095 genes in accordance with their sensitivity to chemical exposures (number of chemical–gene interactions) and level of research attention (publications/gene). (**B**–**D**) Representative GSEA enrichment plots illustrating enrichment of lipid metabolism (**B**), amino acid metabolism (**C**), and glucose metabolism (**D**) with chemically sensitive underexplored genes. (**E**) Biological categories enriched with chemically sensitive underexplored genes identified by ShinyGO 8.0.

The overlap between the CGI/gene dataset and publication numbers/gene dataset consisted of 16,095 genes. Out of this list, 1333 (8.3%) and 14,768 (91.7%) genes had high (≥73 CGIs) and low (<73 CGIs) sensitivity to chemical exposures, respectively; and 555 (3.5%) and 15,540 (96.5%) genes were well explored (≥200 publications/gene) and underexplored (<200 publications/gene), respectively. Among 1333 chemically sensitive genes, 223 (16.7%) were well explored and 1110 (83.3%) genes were underexplored. The distribution of chemical sensitivities vs. the number of publications per gene is shown in Figure 1A and Supplemental Table S1.

The GSEA conducted for the genes highly sensitive to chemical exposures against three databases of biological pathways/categories retrieved coherent results demonstrating that chemically sensitive underexplored genes are enriched significantly with metabolic categories. Categories that were enriched with FDR q < 0.1 are shown in Table 1. Specifically, a range of biological categories related to lipid metabolism was significantly enriched. Enriched categories also included the metabolism of amino acids, glucose, and nucleosides (see Figure 1B–D for representative enrichment plots). Similarly, the GSEA conducted for the underexplored genes insensitive to chemical exposures showed coherent results and demonstrated the significant enrichment of categories related to translation (including mitochondrial translation), ribosomes, and tRNA synthesis, processing, and function (Supplemental Table S2).

**Figure 2:**
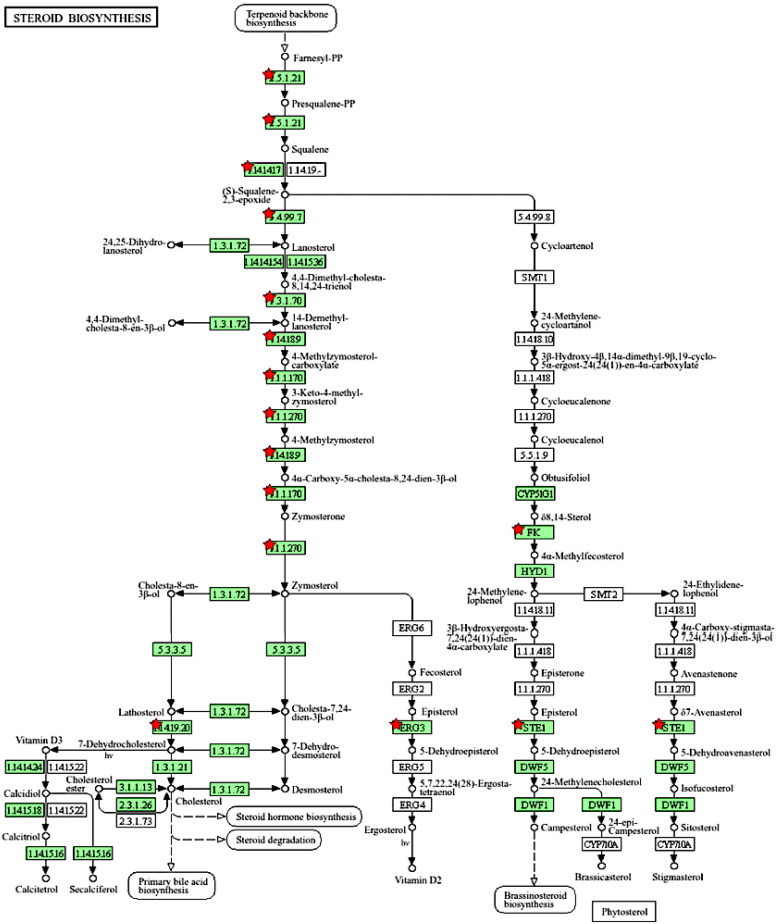
Highly chemically sensitive underexplored genes (red stars) in steroid biosynthesis pathway. Adapted from Kyoto Encyclopedia of Genes and Genomes [27], with permission from Kanehisa Laboratories, 2024.

**Figure 3:**
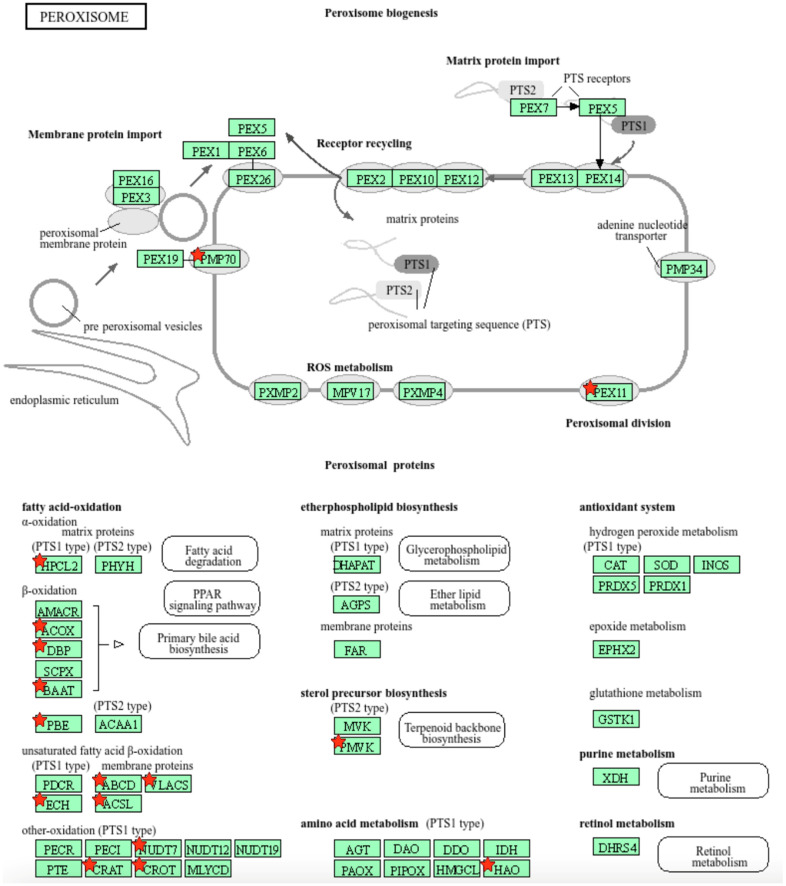
Highly chemically sensitive underexplored genes (red stars) in peroxisome pathway. Adapted from Kyoto Encyclopedia of Genes and Genomes [27], with permission from Kanehisa Laboratories, 2024.

**Figure 4:**
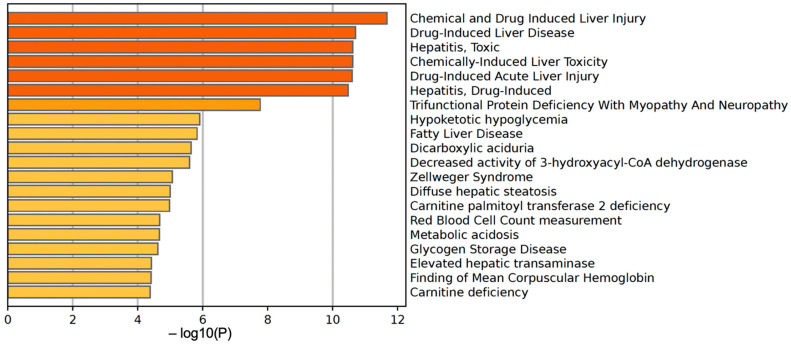
DisGeNET disease categories enriched with highly chemically sensitive underexplored genes.

We further used the shortlist of chemically sensitive genes selected based on a stringent criterion for the level of knowledge availability (≤20 publications/gene) to identify enriched categories using ShinyGO 8.0. The results of this analysis (Figure 1E) confirm that underexplored chemically sensitive genes enrich metabolic pathways, including the metabolism of lipids, amino acids, and glucose, along with categories representing the existing focus of toxicology (e.g., glutathione metabolism and cytochrome P450). The same list of genes was also analyzed using DAVID Functional Annotation Clustering. Concordant with other analyses, the top enriched clusters included categories associated with lipid metabolism (e.g., cluster 1: fatty acid beta-oxidation and peroxisome, enrichment score (ES) = 8.76; cluster 2: lipid and steroid biosynthesis, ES = 8.66), amino acid metabolism (e.g., cluster 5: valine, leucine, and isoleucine degradation; tryptophan metabolism; and lysine degradation, ES = 4.63), and drug metabolism and oxidative stress response (e.g., cluster 3: flavin adenine dinucleotide, ES = 5.31; cluster 6: glutathione metabolism and cytochrome P450, ES = 4.34). To illustrate the role of chemically sensitive underexplored genes in lipid metabolism pathways, their positions in the steroid biosynthesis pathway and peroxisome pathway are shown in Figure 2 and Figure 3, respectively. The full details of the enriched DAVID clusters are shown in Supplemental Table S3. Finally, the analysis of DisGeNET disease categories enriched with underexplored highly chemically sensitive genes retrieved a range of conditions related to drug-induced liver injury, hepatic steatosis, and other pathology of lipid and carbohydrate metabolism (Figure 4).

## 4. Discussion

Our results demonstrate that metabolic pathways, especially the metabolism of fatty acids and amino acids, and to a lesser degree the metabolism of glucose, are underexplored molecular mechanisms sensitive to chemical exposures. These results are concordant with the traditional structure of toxicology. The field of “metabolic disruption” started to take shape only recently. Indeed, the term “metabolic disruption” was first proposed by Casals-Casas and Desvergne in 2011 [35] and it was further promoted by a group of multidisciplinary experts in 2015 [36]. A PubMed search conducted on 16 May 2024 with the key word “metabolic toxicity” retrieved only 247 studies. For comparison, searches with the key words “endocrine disruption”, “reproductive toxicity”, “neurotoxicity”, and “carcinogenicity” resulted in 4273, 4944, 91,356, and 228,470 studies. The Society of Toxicology does not have a specialty section in its structure focusing on metabolic toxicity and most toxicological textbooks do not have chapters focusing on metabolic toxicity as well. The importance of the focus of future efforts on the identification of metabolic molecular mechanisms affected by chemical exposures is dictated by the current epidemic of metabolic disease, one of the biggest public health issues in the modern day [37,38,39,40,41].

Specifically, the disruption of lipid metabolism is central to a range of metabolic and cardiovascular disorders including hepatic steatosis, obesity, metabolic syndrome, hyperlipidemia, and atherosclerosis which in turn is the major risk factor for heart attack, stroke, and vascular dementia. For example, in the United States today, nonalcoholic fatty liver disease (NAFLD) is the most common form of chronic liver disease with 33–88% prevalence [42,43,44], about 30% of Americans have metabolic syndrome [38]. The role of chemical exposures in the rise of these conditions is poorly understood, while better identification of the environmental and dietary risk factors may open opportunities for interventions that due to the prevalence of metabolic conditions may have a tremendous effect on the overall public health. The reanalysis of the existing data accumulated by experimental toxicology provides the first step for the unbiased identification of biological mechanisms and health conditions sensitive to chemical exposures [9,45].

Although this study focuses on genes common to humans and the major medico-biological model species, mice and rats, our findings hold relevance for other species. Indeed, we share a significant number of genes with other organisms. For example, according to the euGenes database, we share around 86–94% percent of genes with other mammalian species, around 80% with chicken, around 75% with fish species, 40–50% with insects, 25% with nematode worms, 30% with yeast, and 9% with rice [46]. Additionally, research demonstrates that metabolic disruption well documented in the human population is not unique to humans, and other species experience similar changes in metabolism [47]. For example, body weights increased over the past several decades in primates and rodents living in research colonies, in feral rodents, and domestic dogs and cats [47]. These findings remain unexplained, although the role of environmental exposures is suspected [48,49]. Our data suggest that at least partly, the adverse metabolic trends in humans and wildlife remain unexplained due to the low level of attention to the relevant molecular mechanisms from the toxicological community.

The major limitation of this study consists of the use of existing gene annotations to identify molecular mechanisms associated with underexplored genes. This approach does not allow the identification of toxicological mechanisms that are not present in the current annotations of underexplored genes. It is reasonable to assume that due to the underexplored nature of these genes, their annotations are far from being complete, and future research may identify other functions of currently underexplored genes. Another limitation is that our crude approach does not allow the identification of the role of each gene in the development of toxicity outcomes. Some chemically sensitive genes may be causally involved in toxicities, others may be involved in compensatory responses, and some other genes may be involved in neither but represent a side effect of AOP or compensatory mechanism activation. Despite these limitations, we suggest that the current analysis identifies the underexplored areas of toxicology. Additional research efforts in this area may provide significant progress and important discoveries.

## Figures and Tables

**Table 1:** Biological categories enriched with chemically sensitive underexplored genes as identified with GSEA against Reactome, KEGG, and Gene Ontology databases (NES–normalized enrichment score; FDR–false discovery rate).

Biological Category	NES	FDR q
**Reactome**		
Glycerophospholipid biosynthesis	−2.16	0.014
The metabolism of amino acids and derivatives	−2.15	0.007
Peroxisomal protein import	−2.06	0.009
Cholesterol biosynthesis	−2.06	0.007
The metabolism of steroids	−1.94	0.018
Fatty acid metabolism	−1.82	0.046
The activation of gene expression by SREBF SREBP	−1.79	0.048
**KEGG**		
Butanoate metabolism	−2.03	0.007
Valine leucine and isoleucine degradation	−2.02	0.004
Fatty acid metabolism	−1.85	0.017
Peroxisome	−1.65	0.062
Glycolysis gluconeogenesis	−1.59	0.073
**Gene Ontology (Biological Process)**		
Organic acid catabolic process	−2.33	0.004
Monocarboxylic acid catabolic process	−2.12	0.016
Fatty acid catabolic process	−2.03	0.028
Fatty acid beta-oxidation	−1.98	0.037
Fatty acid derivative metabolic process	−1.93	0.05
Nucleoside bisphosphate metabolic process	−1.93	0.044
Amino acid metabolic process	−1.85	0.069
Cellular modified amino acid metabolic process	−1.83	0.069
Lipid oxidation	−1.83	0.065
Thioester metabolic process	−1.81	0.072
Alpha amino acid metabolic process	−1.78	0.08
**Gene Ontology (Molecular Function)**		
Oxidoreductase activity, acting on CH-OH group of donors	−2.30	0.003
Oxidoreductase activity, acting on CH-CH group of donors	−1.93	0.038

## Data Availability

All the data on the chemical–gene interactions per gene used in this study are available through Mendeley Data [20]. All the data on the PubMed publications numbers per gene used in this study are available through the Find My Understudied Genes (FMUG) database (https://fmug.amaral.northwestern.edu/, accessed on 2 July 2024).

## Supplementary Materials

The following supporting information can be downloaded at: https://www.mdpi.com/article/10.3390/jox14030052/s1, Table S1: Number of chemical–gene interactions (CGIs) and publications per gene; Table S2: KEGG, Reactome, and Gene Otology categories enriched with chemically insensitive (CGI < 73) underexplored (<200 publications/gene) genes; Table S3: DAVID Functional Annotation Clustering for top underexplored genes (<20 publications/gene) highly sensitive to chemical exposures (CGI > 72).
